# AHNAK suppresses ovarian cancer progression through the Wnt/β-catenin signaling pathway

**DOI:** 10.18632/aging.203473

**Published:** 2021-10-23

**Authors:** Yanlin Cai, Yi Hu, Furong Yu, Wenjuan Tong, Shufen Wang, Shunliang Sheng, Jiayu Zhu

**Affiliations:** 1 Department of Gynecology and Obstetrics, The First Affiliated Hospital of University of South China, Hengyang, Hunan, China; 2 Department of Obstetrics and Gynecology, Nanfang Hospital of Southern Medical University, Guangzhou, Guangdong, China

**Keywords:** AHNAK, metastasis, ovarian cancer, proliferation, Wnt/β-catenin

## Abstract

Globally, ovarian cancer is the 2^nd^ most frequent cause of gynecologic-associated cancer fatalities among women. It has an unfavorable prognosis. There is a need to elucidate on the mechanisms involved in ovarian cancer progression and to identify novel cancer targets. We investigated and verified AHNAK contents in ovarian cancer tissues and corresponding healthy tissues. Then, we overexpressed AHNAK *in vitro* and *in vivo* to establish the roles of AHNAK in ovarian cancer cell proliferation and metastasis. Finally, we evaluated the possible molecular mechanisms underlying. We established that AHNAK was downregulated in ovarian cancer. Elevated AHNAK contents in ovarian cancer cell lines remarkably repressed ovarian cancer cell growth, along with metastasis *in vitro*, as well as *in vivo*. Moreover, AHNAK suppressed the progress of ovarian cancer partly via dampening the Canonical Wnt cascade. Therefore, AHNAK may be a biomarker and treatment target for ovarian cancer.

## INTRODUCTION

Ovarian cancer, which is among the most frequent gynecologic malignancies, is the second most common cause of mortality among women with gynecologic cancers [1]. In 2018 alone, there were 295,414 new morbidities accompanied by 184,799 mortalities from ovarian cancer around the world [2]. Thus far, the prognosis of ovarian cancer is still poor. Elucidating the molecular mechanisms, which underlie the progress of ovarian cancer and developing new targets are needed for better ovarian cancer treatment.

The contents of AHNAK are decreased in cancers, and AHNAK negatively modulates cell growth, as well as work as a tumor repressor via potentiation of TGFβ signaling [3, 4]. Moreover, studies have recently found that AHNAK is pivotal in cell migration along with infiltration in an extensive range of cancers [5]. The knockdown of AHNAK resulted in reduced the dynamics of actin cytoskeleton and activation of MET (mesenchymal-epithelial transition) [6]. Besides, it is believed that the Canonical Wnt cascade is linked to cancer progress, especially with cell migration along with infiltration [7]. Canonical Wnt is recognized to accelerate EMT. Canonical Wnt is hyper-activated in metastatic breast cancer cells [8]. In colorectal cancer, the Canonical Wnt cascade participates in enhanced EMT, cell migration, and cancer cell metastasis [9]. Nevertheless, the function of AHNAK and Canonical Wnt cascade in ovarian cancer is poorly characterized at present.

Herein, we investigated and verified the content of AHNAK in ovarian cancer tissues and corresponding healthy tissues. We demonstrated that AHNAK content was lower in ovarian cancer in contrast with the non-malignant tissues. Elevated levels of AHNAK dramatically repressed ovarian cancer cell progression and metastasis *in vitro* as well as *in vivo*. Moreover, AHNAK suppressed the progress of ovarian cancer partly via inactivating the Canonical Wnt cascade.

## RESULTS

### AHNAK is downregulated in ovarian cancer

**Figure 1:**
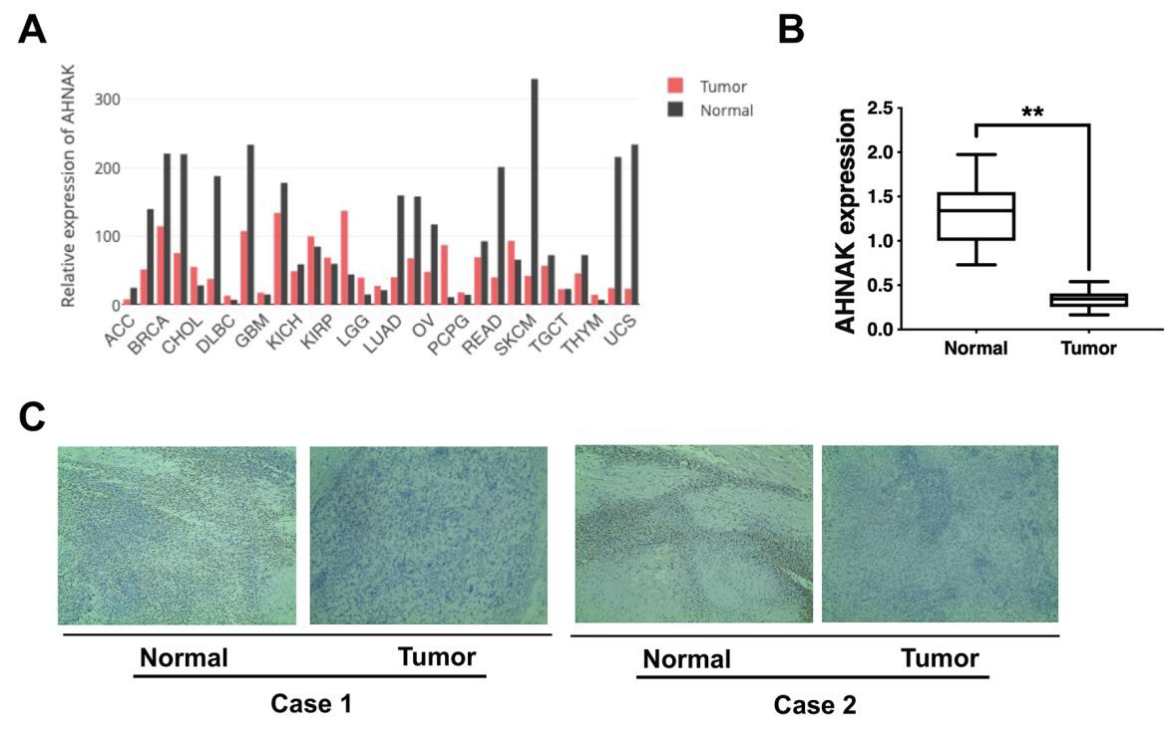
**AHNAK is downregulated in ovarian cancer.** (**A**) AHNAK expression pattern across all tumor samples and matched non-malignant samples. (**B**) AHNAK expression levels in 30 serous ovarian cancer tissues and paired non-malignant tissues. **p < 0.01. (**C**) Images illustrating immunohistochemical staining of AHNAK content in two pairs of paired ovarian cancer samples and their neighboring non-malignant tissue. Original magnification, 200X.

Gene Expression Profiling Interactive Analysis illustrated that AHNAK was downregulated in most cancers, including ovarian cancer (Figure 1A). To verify further AHNAK content in ovarian cancer, we collected 30 tumor serous ovarian cancer tissues and neighboring non-malignant tissues. We detected AHNAK content via qRT-PCR along with immunohistochemistry. The results illustrated that AHNAK was downregulated in ovarian cancer tissues (Figure 1B, 1C), indicating that AHNAK could be a tumor suppressor and could play a vital role in ovarian cancer progress.

### AHNAK overexpression represses ovarian cancer cell growth and infiltration *in vitro*

**Figure 2:**
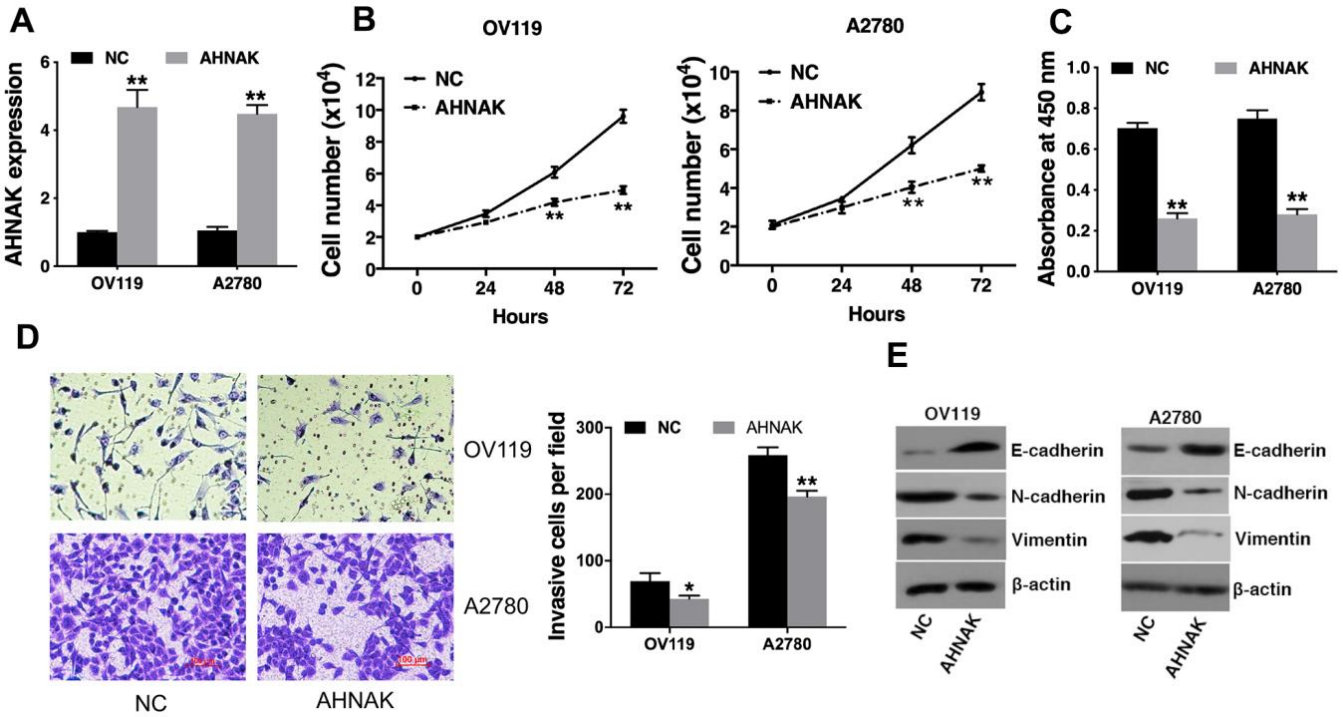
**Elevated AHNAK content represses ovarian cancer cell proliferation along with infiltration *in vitro*.** (**A**) qRT-PCR illustrated that AHNAK was successfully overexpressed. (**B**) Cell proliferation analysis was performed. Cell numbers were evaluated at 24 h, 48 h, and at 72 h after incubation by Coulter Counter (Beckman Coulter, USA). (**C**) CCK-8 assay was performed. (**D**) Transwell assays were performed (left), after which infiltrating cells were quantified by the Image J software (right). (**E**) Western blotting assessment of the levels of indicated epithelial and mesenchymal markers. Data are presented as the mean ± SD for n=3, *p < 0.05, **p < 0.01.

To determine the significance of AHNAK in the progression of ovarian cancer, AHNAK was overexpressed in ovarian cancer cells (Figure 2A). A cell proliferation assay was conducted, and the results demonstrated that AHNAK overexpression suppressed the cell proliferation of ovarian cancer cells (Figure 2B). Moreover, the CCK-8 data illustrated that AHNAK overexpression dramatically repressed the proliferation of ovarian cancers (Figure 2C). Furthermore, we conducted a Transwell assay to assess the function of AHNAK in infiltration of ovarian cancer cells. The data illustrated that elevated content of AHNAK repressed the infiltration of ovarian cancer cells (Figure 2D). To explore whether AHNAK overexpression triggered MET, we assayed the content of the epithelial, as well as mesenchymal biomarkers. Western blotting revealed that AHNAK overexpression elevated E-cadherin levels and suppressed N-cadherin and vimentin levels (Figure 2E).

### AHNAK overexpression suppresses ovarian cancer tumor growth along with migration *in vivo*

**Figure 3:**
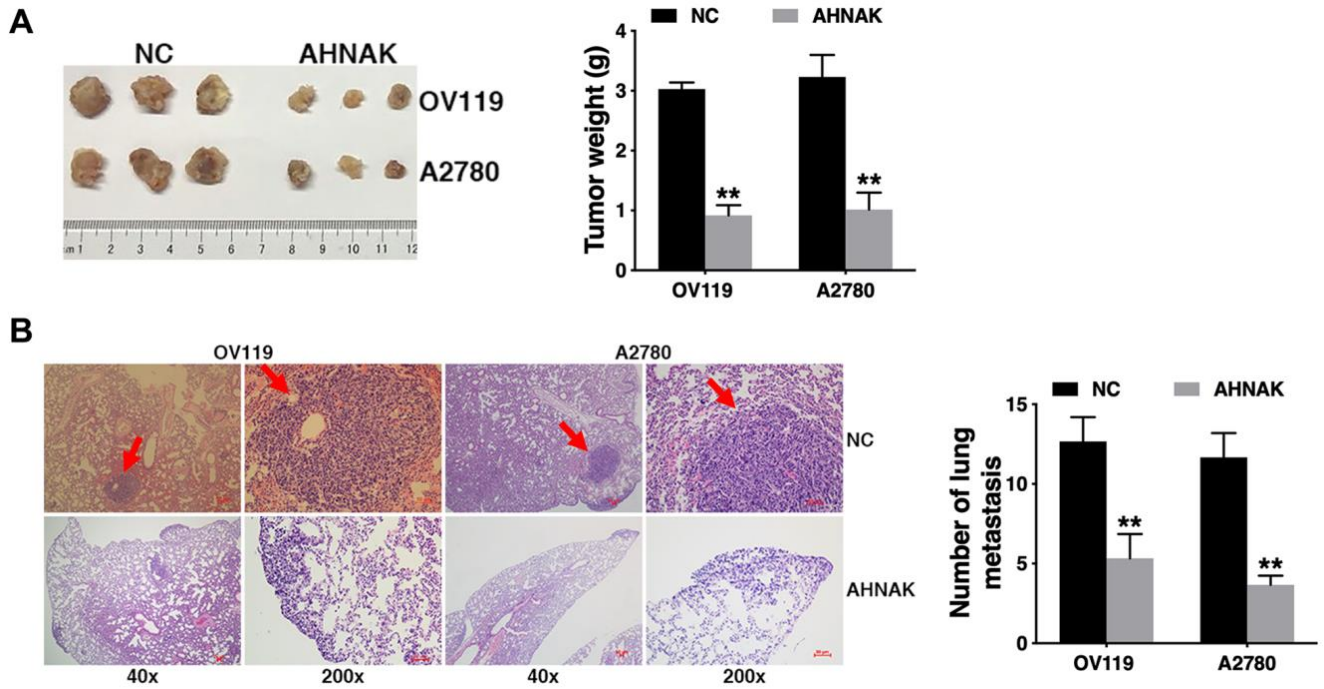
**Elevated AHNAK content suppresses ovarian cancer tumor growth along with migration *in vivo*.** (**A**) The tumors from the mouse xenograft model (n = 3 per group) are shown (left). The weights of the tumors are summarized (right). (**B**) HE-stained sections derived from lung metastatic nodules of the mouse xenograft model (n = 3 per group) are illustrated (left). The number of lung nodules was quantified (right). Original magnification: 40X and 200X, **P < 0.01.

To further assess the biological impacts of AHNAK on ovarian cancer *in vivo*, we established a mouse xenograft model. We found that AHNAK overexpression remarkably diminished tumor growth (Figure 3A), as well as lung metastasis, as illustrated in Figure 3B. The results indicated that AHNAK overexpression represses tumor growth along with migration in ovarian cancer.

### AHNAK represses ovarian cancer progression through the Wnt/β-catenin cascade

**Figure 4:**
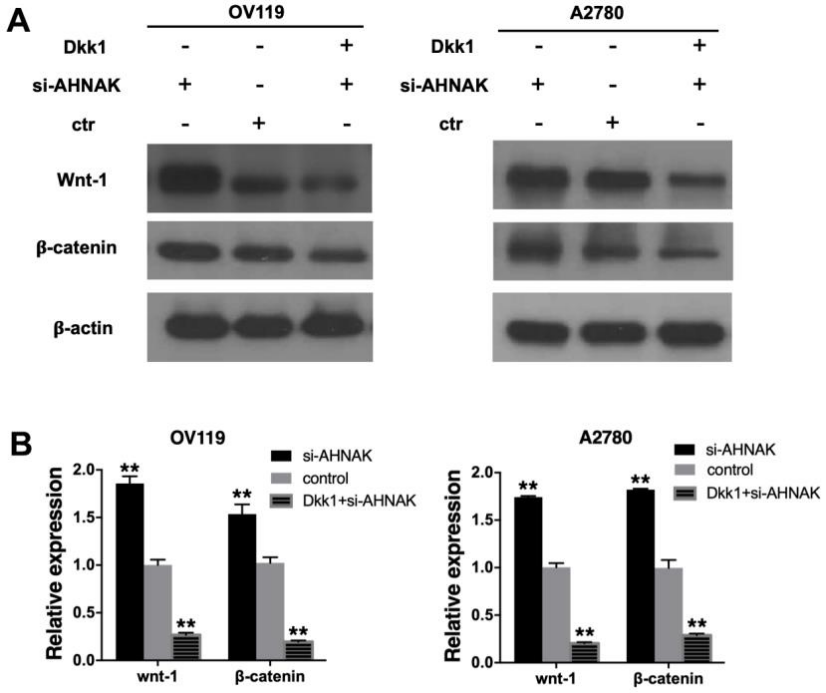
**AHNAK represses ovarian cancer progress by targeting the Wnt/β-catenin cascade.** (**A**) Western blotting evaluation of the contents of Canonical Wnt cascade markers. The Western blotting image in the right panel exhibits that the Wnt inhibitor, DKK1, suppressed Wnt signaling of AHNAK-siRNA activated signal in OV119 and A2780 cells. (**B**) qRT-PCR assays of the contents of Canonical Wnt cascade markers. Data are presented as the mean ± SD for n=3, **p < 0.01.

On the basis of the results of the above experiments, we further assessed the possible molecular mechanisms through which AHNAK exerts its functions in ovarian cancer. It has been documented that the aberrantly activated Canonical Wnt cascade participates in development of cancer [10], and Wnt–β-catenin is correlated with cancer metastasis [11]. Therefore, we investigated whether AHNAK functioned via the Canonical Wnt cascade. The Canonical Wnt axis inhibitor Dkk1 was employed to confirm the function of AHNAK in activating canonical Wnt cascade. The content of wnt-1, β-catenin, and β-actin proteins in si-AHNAK cells was remarkably elevated in contrast with the controls, and Dkk1 effectively repressed this effect. Western blotting (Figure 4A) and qRT-PCR (Figure 4B) revealed that AHNAK knockdown increased the contents of Canonical Wnt cascade markers, indicating that AHNAK functioned partly via modulating the Canonical Wnt axis.

## DISCUSSION

Globally, ovarian cancer has been reported to be the second most common cause of gynecologic-associated cancer fatalities among women, with most patients exhibiting metastatic disease at diagnosis. Despite the increasing improvement in surgery, chemotherapy, radiotherapy, and other targeted therapies, the prognosis of ovarian cancer is still unsatisfying [12–14]. Therefore, elucidating the molecular mechanisms underlying ovarian cancer progress and developing new targets are urgently needed.

AHNAK is a protein associated with various cellular processes, e.g., cell migration and infiltration. In prostate cancer, BRD4 regulates cell migration along with infiltration through the transcription of AHNAK [15]. In liver cancer, RNF38 was shown to induce cellular EMT by promoting TGF-β signaling through AHNAK ubiquitination and degradation [16]. Moreover, AHNAK is involved in chemotherapeutic responses. In breast cancer, there is a correlation between AHNAK and resistance to doxorubicin [17]. In non small-cell lung cancer, UBE3C ubiquitinated and promoted AHNAK degradation, resulting in enhanced stemness [18]. AHNAK is downregulated and remarkably linked with poor survival in numerous cancers, such as glioma [19] and melanoma [20]. Engqvist et al. recently reported that AHNAK was one of the most common fusion partners and functioned as a significant driver fusion transcript in early-stage ovarian cancer [21]. Nevertheless, the mechanism of AHNAK in ovarian cancer is still unclear.

Herein, we illustrated that AHNAK was downregulated in ovarian cancer (Figure 1). Further experiments illustrated that AHNAK overexpression dampened ovarian cancer cell proliferation along with infiltration *in vitro* (Figure 2) and suppressed tumor growth and migration *in vivo* (Figure 3).

The above experiments prompted us to examine the possible molecular mechanisms underlying the biological functions of AHNAK in ovarian cancer. Previous research documented that the Canonical Wnt cascade is related with the progress of cancer, especially with cell migration and infiltration [7–9]. The Canonical Wnt cascade promotes cancer progress in ovarian cancer through multiple mechanisms including EMT, cancer stemness, as well as therapy resistance [22]. In ovarian cancer, the regulatory mechanisms for Wnt/β-catenin cascade included gene as well as non-coding RNA alterations, epigenetic alterations, several soluble factors in ovarian cancer ascites, and extracellular vesicles such as exosomes.

Accumulative evidence documented that Canonical Wnt cascade has an indispensable role in metastasis of ovarian cancer through activation of EMT. S Bernaudo et al. reported that cyclin G2 suppressed ovarian cancer via repressing EMT through disrupting the Canonical Wnt cascade [23]. IQGAP2 was reported to inhibit ovarian cancer cell EMT, migration along with infiltration by suppressing the Wnt-induced nuclear translocation of β-catenin, as well as transcription [24]. Ovarian cancer has a unique mechanism of peritoneal migration. There are no anatomical barriers between the primary site and abdominal cavity. EMT is involved in initiation of exfoliated malignant cell dissemination; therefore, increases the metastasis and migration of ovarian cancer. Herein, we found that elevated AHNAK content decreased the quantities of Canonical Wnt cascade markers and epithelial marker β-catenin, illustrating that AHNAK functioned partly via modulation of the Wnt–β-catenin cascade (Figure 4). Besides, our results illustrated that AHNAK overexpression suppressed tumor growth along with migration *in vivo* and *vitro*. Taken together, the results illustrated that AHNAK might inhibit tumor infiltration and migration by repressing EMT via modulating the Canonical Wnt cascade.

In summary, AHNAK levels in ovarian cancer are suppressed while AHNAK overexpression inhibits ovarian cancer progression by targeting the Canonical Wnt cascade. Therefore, AHNAK might be a marker, as well as treatment target for ovarian cancer. However, there were some limitations in our study. One limitation is, for instance, the fact that other functions of AHNAK in cancer and its relevance to the current research are not described: for example, the role of AHNAK in EMT. Also, more clinical data would increase the validity of the results. Hence, further investigation of the underlying mechanism is warranted.

## MATERIALS AND METHODS

### Gene expression of AHNAK in GEPIA

GEPIA (http://gepia.cancer-pku.cn/index.html), a web server for assessing RNA expression data for 9,736 cancers and 8,587 non-malignant samples from TGGA along with the Genotype-Tissue Expression projects. The expression profile of AHNAK in different cancers was explored in GEPIA.

### Clinical samples and immunohistochemistry

Thirty paired serous ovarian tumor and neighboring non-malignant tissues were acquired from The First Affiliated Hospital of the University of South China. The immunohistochemistry (IHC) of AHNAK staining (1:250, Abcam, USA) was performed as described previously [5]. Certified pathologists evaluated the staining in a blinded manner.

### Cell growth and transfection

Ovarian cancer OV119 and A2780 cell lines were obtained from the American Type Culture Collection (USA) and were grown as described by the manufacturer. Cells were validated to be mycoplasma infection-free, and the authenticity was confirmed via DNA fingerprinting.

For AHNAK overexpression experiments, an AHNAK-expressing lentivirus vector (EX-V0190-Lv122) as well as control plasmid (EX-NEG-Lv122) were provided by GeneCopoeia (USA). The Lenti-Pac^™^ HIV Expression Packaging Kit (Cat No, GeneCopoeia) was employed to generate lentiviruses. Lentiviral cell infections were performed using Lipofectamine 2000 (Cat No, Invitrogen, USA) and screened with puromycin (2 μg/mL) to obtain AHNAK-expressing cells. For AHNAK knockdown experiments, cells were inserted with si-AHNAK (Santa Cruz Biotechnology, sc-97060) via transfection as described by the manufacturer. A nontargeting siRNA was used as control. Then, the cells were grown and utilized in the subsequent assays.

### Quantitative RT-PCR (qRT-PCR) analysis

RNA extraction was performed using the TRIzol reagent (Invitrogen). Denaturing agarose gel was employed to check RNA integrity via electrophoresis. PrimeScript^TM^ RT Master Mix as well as SYBR^®^ Premix Ex Taq^TM^ II (Cat No, Takara, Japan) were used to perform qRT-PCR using the Bio-Rad CFX96 PCR System (USA). Primers used in this study were synthesized by Invitrogen and they are shown in Supplementary Table 1. β-actin value served as the normalization standard and calculated by the 2^-ΔΔCt^ approach. Each reaction was repeated in triplicates.

### Cell proliferation assay

OV119 and A2780 cells (2 × 10^4^) were planted, and we counted the numbers of cells at 24 h, 48 h, and at 72 h post incubation using the Coulter Counter (Beckman Coulter, USA).

### CCK-8 assay

OV119 and A2780 cells (1 × 10^3^) inoculated at 37° C for 24h prior to transfection. Then, cells were incubated for another 48 h after which the CCK-8 solution (Cat No, Dojindo Laboratories, Japan) was introduced. Cells were incubated for 2 h at 37° C after which absorbance at 450 nM was read using a microplate reader.

### Transwell assay

OV119 and A2780 cells (1 × 10^4^) were cultured, and a medium supplemented with 10% FBS introduced in the lower compartment (BD Biosciences, USA) as a chemoattractant. Afterwards, we fixed the cells with methanol, followed by 0.1% crystal violet staining, and counting.

### Wnt treatment and western blot assay

OV119 and A2780 cells were cultured with or without the addition of the Wnt–β-catenin cascade inhibitor Dkk1 (100 ng/ml, R&D Systems). Proteins of OV119 and A2780 were extracted, and then the concentration determined, fractionated by SDS-PAGE gel (10%) and transfer-embedded to PVDF membranes (Millipore, USA). Then, membranes were blocked with 5% skim milk for 1 h at room temperature. Then the membranes were inoculated with primary antibodies against E-cadherin (Cell Signaling Technology, USA; 1:1000), N-cadherin (Cell Signaling Technology; 1:1000), vimentin (Cell Signaling Technology; 1:1000), wnt-1 (Abcam, USA; 1:1000), β-catenin (Cell Signaling Technology; 1:1000), as well as c-myc (Cell Signaling Technology; 1:1000). Then, an HRP-labeled secondary antibody (Cell Signaling Technology) was added. The anti-β-actin antibody (Affinity, USA; 1:1000) was used as the control.

### Mouse xenograft model

The animal study was performed as previously described [25, 26]. OV119 and A2780 cells were stably transfected with AHNAK or vector and suspended in PBS at 1 × 10^7^ cells/ml, respectively. Then, cells (2 × 10^6^) were subcutaneously inoculated into 4 week old female BALB/c nude mice using a 1-ml injector. (n = 3 per group to provide a power of 90% for a remarkable level of 0.05 with a two-tailed t-test). After 4 weeks, mice were anesthetized after which xenograft tumors were excised and tumor weights determined.

For lung metastasis experiments, OV119 and A2780 cells (1 × 10^5^) were administered through the tail vein by using a microsyringe (n = 3 per group to provide a power of 90% for a remarkable level of 0.05 with a two-tailed t-test). Cells (2 × 10^6^ cells/ml) were dispersed in PBS. After 8 weeks, mice were anesthetized after which lungs were excised. The number of lung macro metastatic nodules were counted and verified by hematoxylin and eosin (HE) staining, which was explored via certified pathologists in a blinded manner.

### Statistical analysis

Statistical analyses were implemented using the SPSS 19.0 software. Between group comparisons were performed using t-tests. Data are presented as the mean ± SD for three independent experiments, unless otherwise stated. Differences were considered statistically significant when *p* ≤ 0.05.

### Data accessibility statement

The data supporting the findings in this study are available from the corresponding author upon reasonable request.

### Ethics committee approval and patient consent

Ethical approval for this study was obtained from the Ethics Committee of The First Affiliated Hospital of the University of South China. Experiments were performed according to the ethical standards formulated in the Declaration of Helsinki. Informed consents were obtained from all patients. The use of animals in this study was approved by the Institutional Research Ethics Committee of The First Affiliated Hospital of the University of South China and performed according to institutional guidelines.

## Supplementary Material

Supplementary Table 1
