# Supplementary material for: AHNAK suppresses ovarian cancer progression through the Wnt/β-catenin signaling pathway
Source: Aging (Albany NY). 2021 Oct 23;13(20):23579–87. doi: 10.18632/aging.203473 (PMC8580348; doi:10.18632/aging.203473)
Supplement: Supplementary Table 1 [file aging-13-203473-s001.pdf]

SUPPLEMENTARY TABLE

Supplementary Table 1. Oligonucleotide sequences for primers used in this study.

Construct	Species	Direction	Sequence (5' - 3')
AHNAK	Human	Forward	ATGCTCCAGGGCTCAACCT
		Reverse	CGTGCCCCAACGTTAAGCTT
wnt1	Human	Forward	ATGGGGCTCTGGGCGCTGTTG
		Reverse	TCACAGACACTCGTGCAGTAC
β-catenin	Human	Forward	CCGCATGGAAGAAATAGTTGAAG
		Reverse	CAATTCGGTTGTGAACATCCC
c-myc	Human	Forward	AGAAATGTCCTGAGCAATCACC
		Reverse	AAGGTTGTGAGGTTGCATTTGA
β-actin	Human	Forward	AGCGAGCATCCCCCAAAGTT
		Reverse	GGGCACGAAGGCTCATCATT
